# Nevirapine versus efavirenz-based antiretroviral therapy regimens in antiretroviral-naive patients with HIV and tuberculosis infections in India: a pilot study

**DOI:** 10.1186/1471-2334-13-482

**Published:** 2013-10-17

**Authors:** Sanjeev Sinha, Puroshottam Raghunandan, Rahul Chandrashekhar, Surendra K Sharma, Sanjiv Kumar, Sahajal Dhooria, Meera Ekka, Thirumurthy Velpandian, Sanjay Ranjan, Hafeez Ahmad, Jyotish Chandra Samantaray, Srinivasaraghavan Venkatesh, Bharat Bhushan Rewari, Nawaid Hussain Khan, Ravindra Mohan Pandey

**Affiliations:** 1 Departments of Medicine, All India Institute of Medical Sciences, Ansari Nagar, New Delhi 110029, India; 2 Ocular Pharmacology & Pharmacy, All India Institute of Medical Sciences, Ansari Nagar, New Delhi 110029, India; 3 Microbilogy, All India Institute of Medical Sciences, Ansari Nagar, New Delhi, 110029 India; 4 National AIDS Control Organisation, Ministry of Health & FW, Government of India, New Delhi 110001, India; 5 Biostatistics, All India Institute of Medical Sciences, Ansari Nagar, New Delhi 110029, India

**Keywords:** Antiretroviral, Efavirenz, HIV, Nevirapine, Tuberculosis

## Abstract

**Background:**

Administration of rifampicin along with nevirapine reduces the plasma concentration of nevirapine in human immunodeficiency virus positive individuals with concomitant tuberculosis (HIV-TB patients). Nevirapine is a much cheaper drug than its alternative efavirenz, and might be beneficial in resource constrained settings.

**Methods:**

A randomised open label trial was conducted at All India Institute of Medical Sciences, New Delhi, India. During the regimen of an antiretroviral therapy (ART), naive HIV-TB patients were randomly assigned to receive either nevirapine or efavirenz based ART with concomitant rifampicin based anti-tubercular therapy (ATT). Participants were followed for 24 months after starting ART. The end points were virological, immunological and clinical responses, and progression of HIV disease marked by failure of ART.

**Results:**

Of the 135 HIV-TB patients, who were receiving rifampicin based ATT, 68 were selected randomly to receive efavirenz based ART and 67 to receive nevirapine based ART. The virological failure rates in the overall population, and the nevirapine and efavirenz groups were 14.1% (19/135); 14.9% (10/67) and 13.2% (9/68), respectively (p = 0.94). No significant difference was found between the groups in the rate of clinical, immunological or virological failures. The overall mortality was 17% with no significant difference between the two groups. Except for the lead in period on day 14, the mean nevirapine concentration remained above 3 mg/L. No association was found between plasma levels of nevirapine and incidence of unfavourable outcomes in this group.

**Conclusions:**

Outcome of ART in HIV-TB patients on rifampicin based ATT showed no significant difference, irrespective of whether efavirenz or nevirapine was used. Therefore, nevirapine based ART could be an alternative in the resource limited settings in patients with HIV and tuberculosis co-infection.

**Trial registration:**

NCT No. 01805258.

## Background

According to the UNAIDS 2010 report on the global AIDS epidemic, there are 33.3 million people living with human immunodeficiency virus/acquired immunodeficiency syndrome (HIV/AIDS) in the world [1]. Furthermore, Tuberculosis (TB) is the most common opportunistic infection affecting around 40% of people with HIV/AIDS worldwide [2]. As per the recent report of National AIDS Control Organisation (NACO), the prevalence of HIV in India is 0.29% with a total burden of 2.27 million HIV-infected patients. However, there has been a decline in the incidence of new HIV/AIDS cases in India after the introduction of a free ART program by NACO since April, 2004 [3]. Anti-retroviral therapy (ART) based on non-nucleoside reverse transcriptase inhibitors (NNRTIs) is a widely used regimen, particularly in resource-limited countries. According to the current WHO HIV treatment guidelines and the NACO guidelines for India, efavirenz-based ART is a preferred first-line regimen in HIV/TB co-infected patients already receiving rifampicin-containing ATT regimen, because of the lower drug-drug interactions when compared with nevirapine or protease inhibitors (PIs) [4,5]. However, nevirapine is frequently used in India in HIV/AIDS patients as a component of first line regimens, which is also available as fixed drug combinations (with zidovudine plus lamivudine or stavudine plus lamivudine). These drug combinations ensure good adherence as they are given as two tablets twice a day, are modestly priced, do not require food restrictions, and are safe during pregnancy. To date, rifampicin, a potent cytochrome P450 enzyme inducer, which reduces the plasma concentrations of nevirapine, is still an important drug prescribed for the treatment of TB in many resource limited countries like India. This drug is usually administered for 6 to 8 months as a part of ATT to reduce the relapse of TB in HIV-TB co-infected patients. Although, rifabutin has fewer problematic drug interactions, it is not available in many resource limited countries including India.

The concomitant use of ART and ATT is associated with a reduction in the mortality in patients co-infected with HIV and tuberculosis. Nevertheless, treatment in this setting is complex because of high pill burden leading to poor adherence, drug-drug interactions and hepatotoxicity caused by both nevirapine and rifampicin. There is also a rising concern that rifampicin, a potent inducer of cytochrome P450 enzyme, reduces the plasma concentrations of nevirapine leading to virological failure and occurrence of resistance mutations [6-8]. However, recent studies have shown that even though nevirapine concentrations are lower when it is co-administered with rifampicin, the immunological and virological responses of nevirapine-containing ART have been found to be satisfactory [9,10].

While multiple studies have shown that both nevirapine and efavirenz based regimens have equal efficacy in ART naive patients without TB, but there is not much information in the literature in setting of HIV-TB co-infection [11,12]. The present study is a comparative, randomised control trial, conducted prospectively to compare the safety and efficacy of nevirapine and efavirenz based ART in HIV-TB co-infected ART-naive patients, who were concomitantly receiving rifampicin based anti-tubercular regimen (ATT). In this study, we have also measured the plasma nevirapine concentrations and correlated them with the immunological and virological responses to ART for a follow up period of more than two years.

## Methods

This was a randomised control study conducted at All India Institute of Medical Sciences (AIIMS), New Delhi, between September, 2007 to December, 2012. Patients, positive for HIV by ELISA, ART-naïve and presented with concomitant TB, were enrolled as study participants. Only patients having CD4 count <200 cells/mm^3^ and with normal renal and hepatic function were included. The other inclusion criteria were age >18 years and absence of concomitant diabetes mellitus. Patients positive in hepatitis B and C serologies were excluded to avoid confusion between drug induced and viral hepatitis. Besides these, patients on anti-epileptic drugs, immunosuppressant and other drugs that induce liver microsomal enzyme systems were also excluded. All female patients were screened with a urine pregnancy test and were excluded if pregnant. HIV infection was documented by licensed ELISA test kit (As per NACO guidelines) [5]. CD4/CD8 cell counts were determined by flow-cytometry (BD FACS CALIBUR). Viral load testing was done using AMPLICOR HIV-1 Monitor Test, version 1 · 5, manufactured by ROCHE Diagnostics and Abbott’s RealTime HIV-1 Qualitative Assay performed on Abbott’s automated high-throughput *m*2000 system. The protocol was approved by the institutional research Ethics Committee of the All India Institute of Medical Sciences, New Delhi. All participants gave signed informed consent to participate in this study.

### Initial evaluation

All patients underwent a detailed physical examination. Their body mass index (BMI) was calculated. Haemoglobin, complete blood counts, erythrocyte sedimentation rate, fasting blood glucose, renal function tests, liver function tests, serum albumin, serum uric acid and routine urinalysis were done for all patients. In addition, their CD4 counts and plasma HIV viral load were also determined.

### Randomisation and treatment

In this randomised open label trial, eligible ART naive HIV-TB patients were assigned to receive either nevirapine or efavirenz based ART. All the ART naive patients attending the ART clinic at our centre were screened for tuberculosis by physical exam, sputum examination for AFB, chest radiographs and ultrasound abdomen as part of routine screening recommended by NACO and Revised National Tuberculosis Control Programme (RNTCP) [5]. ART naive patients co-infected with tuberculosis were randomised into one of the trial arms using computer generated random number tables. ATT was started for the patients according to the RNTCP guidelines for directly observed therapyshort-course (DOTS). After 2–8 weeks of ATT, ART was started, which consisted of zidovudine and lamivudine combined with either twice a day nevirapine or once a day efavirenz as per the respective randomisation. Those who had haemoglobin less than 8 g/dl were administered stavudine in place of zidovudine. The administered doses were in accordance with the NACO guidelines. Zidovudine was given in a dose of 300 mg twice a day, lamivudine 150 mg twice a day and stavudine 30 mg twice a day. Nevirapine was administered at a dose of 200 mg once a day for the first 14 days (called the lead-in dose) as per NACO guideline, and then the dose was escalated to 200 mg twice a day. The patients were advised to take the drug at 9 am for the first 14 days and at 9 am and 9 pm during the rest of the period of the follow up. Efavirenz was given in a dose of 600 mg per day. The timing advised for efavirenz intake was daily at 9 pm after dinner.

### Follow up

Patients were assessed at day 14 after the start of ART, then on day 28, and every 4 weeks thereafter through 96 weeks. A complete haemogram, and liver and kidney function tests were obtained on all of the visits. CD4 counts and HIV plasma viral loads were measured at baseline, 6, 12, 18, and 24 months after the start of ART. Trough nevirapine concentrations were assessed at day 14, 28, 42 and 180, 12 hours after the evening dose of nevirapine in all patients. The method used for the measurement of nevirapine concentrations has been described earlier [10].

### Definitions

Disease progression or clinical failure was defined as a new or recurrent WHO stage-4 condition, after at least 6 months of ART [4]. Immunological failure was defined as a decrease in CD4 count from the baseline values, for that either 50% decrease from the peak CD4 count during the treatment or persistent counts below 100 cells/mm^3^ after 24 weeks of the treatment was considered [4]. Virological response was defined as HIV plasma viral loads <400 copies/ml after 24 weeks of ART [4]. Composite unfavourable outcome was defined as when a patient failed to suppress the HIV plasma viral load to <400 copies/ml at the end of 24 weeks of the treatment, or failed to sustain a suppressed plasma viral load <400 copies/ml after 24 weeks, or had immunological failure at any time during the treatment as defined above, or had the disease progression as defined above, or expired during the treatment. Combined ART failure was defined as the development of clinical, immunological or virological failure at anytime during the treatment. Treatment success and failure of ATT were defined as per the WHO guidelines [13].

### Outcomes

The primary outcome of the study was the proportion of the subjects after 24 months who died or had a CD4 count below 200 cells/ml at 24th month. The secondary outcome of the study was assessment of safety and tolerability of ART, measured by the proportion of the subjects with toxicities and the proportion of subjects changed/discontinued ART because of toxicities or treatment failure. The overall outcome of ATT was assessed by both of the outcome.

### Statistical analysis

Data were recorded on a pre-designed data sheet and managed on an MS-Excel spreadsheet. All entries were checked twice for any possible recording error. Mean, frequency and median were calculated for all quantitative variables along with their respective standard deviations and Interquartile ranges. Initially, considering this a pilot study, sample size calculation was not planned and data were gathered as per the sample of convenience. All analyses were done by Intention to treat analysis principle. All continuous variables having normal distribution were analysed using Student’s *t*-test. Ordinal variables and variables with non-normal distribution were analysed using Wilcoxon rank-sum test. The categorical variables with dichotomous outcomes like ART failure and unfavourable outcomes were analysed using logistic regression model. Generalised estimation equations were used to analyse the predictors of immunological response in terms of the increase in CD4 count. Statistical analyses were performed using software package STATA version 11 · 0 [(intercooled version), Stata Corporation, Houston, Texas, USA].

## Results

**Figure 1:**
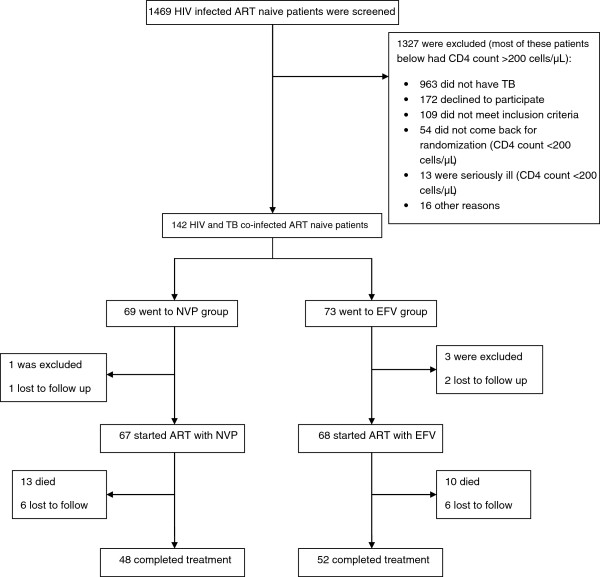
Screening, enrolment and follow-up of study participants.

Of the total 135 HIV-TB patients enrolled, 67 were randomised into nevirapine (NVP) arm and 68 into efavirenz (EFV) arm of the study (Figure 1). Their baseline characteristics are summarised in Table 1. The two groups were not significantly different in any respect; however, the distribution of the type of TB bordered at a nearly significant level (p = 0 · 06), owing to the greater number of disseminated/miliary TB cases in the nevirapine group. Majority of the patients (96 · 3%, 130/135) were at WHO stage-4 of the HIV disease, and the rest were at stage-3. There was a clear male predominance in both the groups in terms of gender distribution. Most of the patients were suffering from their first episode of TB, and therefore, were started on DOTS category I ATT.

**Table 1:** Baseline characteristics of the study participants

**Variable**	**Nevirapine group**	**Efavirenz group**	**P value**
	**n = 67**	**n = 68**	
Age, years:			
Mean ± SD^a^	36.3 ±9.2	34.8 ±6.9	0.29
Gender, number (%):			
Male	53 (79.1)	59 (86.8)	0.24
Female	14 (20.9)	09 (13.2)	
BMI^b^, kg/m^2^:			
Mean ± SD	18.2 ±2.7	18.4 ±2.7	0.57
Haemoglobin, g/dL:			
Mean ± SD	10.0 ±1.9	10.4 ±1.8	0.27
CD4 count, cells/mm^3^:			
Median (Range)	137 (20–506)	139 (7–588)	0.90
log_10_ viral load/ml:			
Median (Range)	5.52 (2.60–6.58)	5.19 (2.76–6.76)	0.43
WHO Staging of HIV disease, number (%):			
Stage-3	03 (4.5)	02 (2.9)	0.64
Stage-4	64 (95.5)	66 (97.1)	
Type of Tuberculosis, number (%):			
PTB^c^	16 (23.9)	19 (27.9)	0.06
EPTB^d^	31 (46.3)	40 (58.8)	
Disseminated/Miliary TB	20 (29.8)	09 (13.3)	
Category of ATT^e^, number (%):			
Category I	52 (77.6)	55 (80.9)	0.64
Category II	15 (22.4)	13 (19.1)	
ATT-ART^f^ gap, days:			
Median (Range)	27 (11–85)	26 (04–93)	0.92

^a^Standard Deviation, ^b^Body Mass Index, ^c^Pulmonary Tuberculosis, ^d^Extra Pulmonary Tuberculosis, ^e^Antituberculosis treatment, ^f^Antiretroviral therapy.

Table 2 presents outcomes of ATT and ART of the participants. The overall mortality was 17% (23/135) with no significant difference between the groups. The mortality rates were 19 · 4% (13/67) and 14 · 7% (10/68) in the nevirapine and efavirenz groups, respectively (p = 0 · 46). Considering the type of TB, the difference in mortality between the groups was further reduced (p = 0 · 50). Of the 23 patients who died, 16 (69 · 6%) had a CD4 count <100 cells/mm^3^. Also, most of the deaths (87%, 20/23), in both the groups, occurred at the early stage of the treatment, when the patients were receiving both ATT and ART. Outcome of TB treatments in both the groups was comparable (p = 0 · 26).

**Table 2:** Outcomes of antituberculosis and antiretroviral treatment


**Outcomes of tuberculosis at completion of ATT**			
**Outcome**	**Nevirapine group**	**Efavirenz group**	**P value**
	**n = 67**	**n = 68**	
Successfully treated	50 (74.6%)	51 (75.0%)	0.26
Lost to follow up	06 (9.0%)	06 (8.8%)	
Died	11 (16.4%)	09 (13.2%)	
On ATT at end of study	00	02 (2.9%)	
**Outcomes of ART after 24 months**			
**Outcome**	**Nevirapine group**	**Efavirenz group**	**P value**
	**n = 67**	**n = 68**	
Mortality:			
Observed	13 (19.4%)	10 (14.7%)	0.46
(% Adjusted for type of TB)	19.1%	14.9%	0.50
ART failure:	19 (28.4%)	21 (30.9%)	0.75
Clinical failure	06 (9.0%)	06 (8.8%)	0.98
Immunological failure	06 (9.0%)	08 (11.8%)	0.58
Virological failure	10 (14.9%)	09 (13.2%)	0.94
Composite unfavourable outcome	30 (44.8%)	29 (42.6%)	0.98
(Death and/or ART failure)			

*ATT* Antituberculosis treatment, *ART* Antiretroviral therapy.

**Figure 2:**
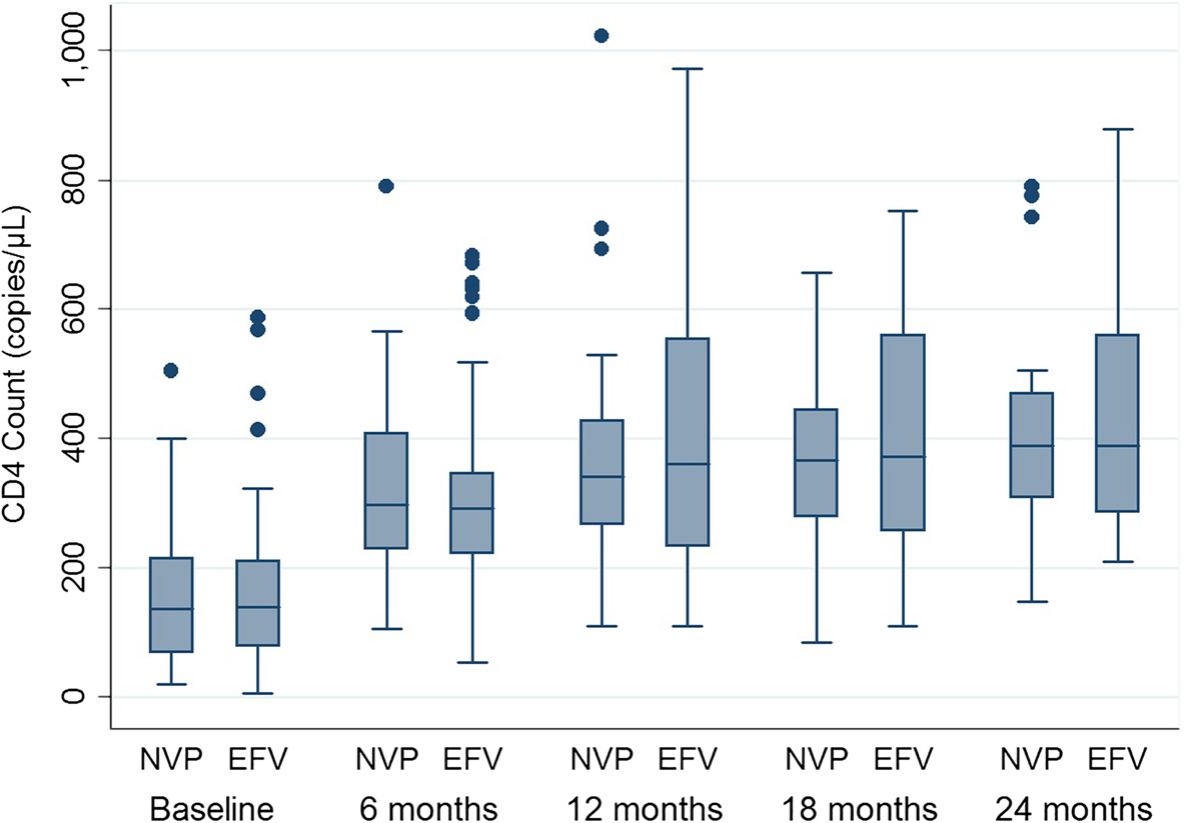
**CD4 cell count at different time points in nevirapine and efavirenz groups.***NVP* nevirapine, *EFV* efavirenz.

**Figure 3:**
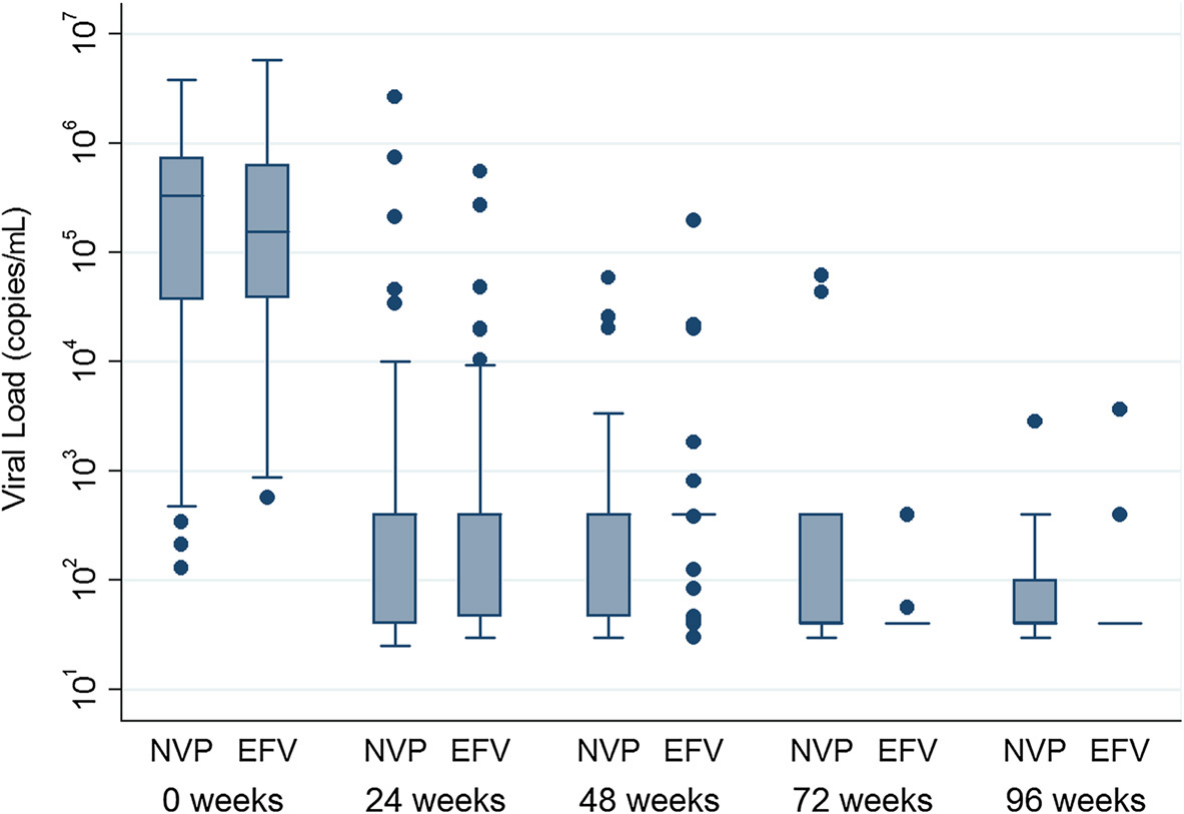
**Viral load count at different time points in log scale in nevirapine and efavirenz group.***NVP* nevirapine, *EFV* efavirenz.

**Figure 4:**
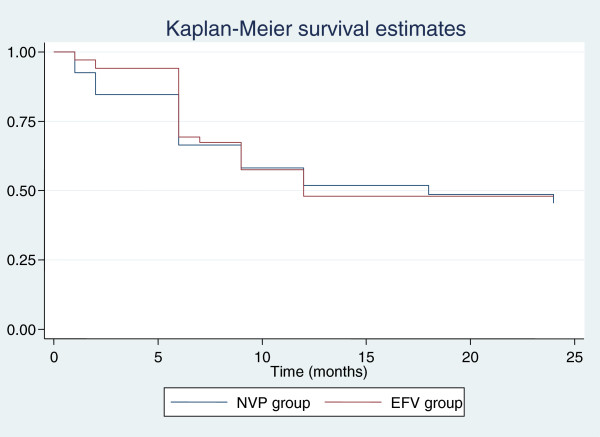
**Kaplan Meier survival curve with cumulative probability of death or ART failure by 24 months.***NVP* nevirapine, *EFV* efavirenz.

The combined incidence of ART failure was 28 · 4% (19/67) in the nevirapine arm and 30 · 9% (21/68) in the efavirenz arm (p = 0 · 75) with an overall ART failure of 29 · 6% (40/135). Rates of clinical, immunological and virological types of failures were compared separately, which reflected no significant difference between the groups for any category (Figures 2, 3). Composite unfavourable outcome was defined as either death or any type of ART failure or both (Figure 4). The incidence of composite outcome in the entire study population was 43 · 7% (59/135). Nevirapine group had 44 · 8% (30/67) composite unfavourable outcome, and efavirenz group had 42 · 6% (29/68); p = 0 · 98.

**Figure 5:**
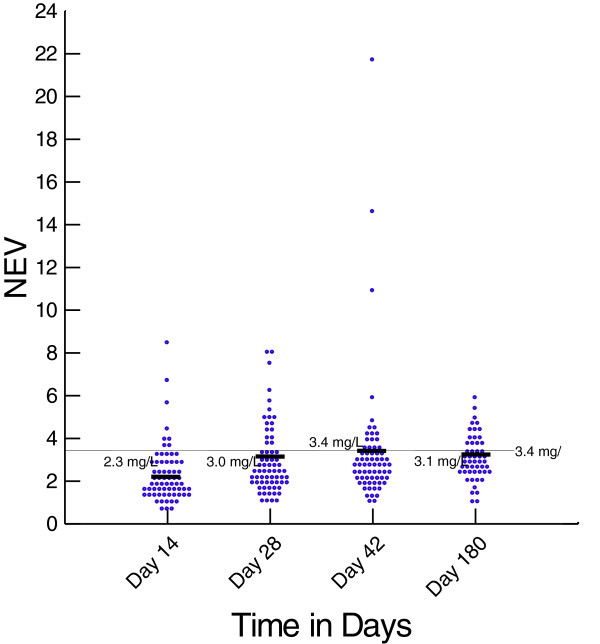
Plasma Nevirapine concentrations at different time points in nevirapine group.

Nevirapine drug level was measured in the stored plasma collected from patients in the arm on day 14, 28, 42, and 180 after the initiation of ART. Except for the lead in period on day 14, the mean nevirapine concentration remained above 3 μg/mL (Figure 5). The mean difference in the plasma nevirapine concentrations between those who had composite unfavourable outcome at 24th month as compared to those who had favourable outcome were 0.01 (P = 0.97) at day 14, 0.21(P = 0.11) at day 28, 0.34 (P = 0.13) at day 42 and 0.62 (P = 0.06) at day 180. No association was found between plasma levels of nevirapine and incidence of unfavourable outcomes in this group at any time-point.

The rate of adverse events was similar in the two groups. Seven patients in the nevirapine arm and six in the efavirenz arm needed a change in the ART regimen after developing an adverse effect to one of the NRTI constituents, zidovudine or stavudine. No regimen change was required with respect to the NNRTI component of ART. No hospital admission or death was ascribed to any adverse event.

General health parameters like body mass index, blood haemoglobin level, and liver function tests showed similar favourable trends over time in both the groups (Table 3).

**Table 3:** Blood parameters and BMI at different time points

**Variable mean ± S.D.**	**Nevirapine group (n = 67)**			**Efavirenz group (n = 68)**		
	**Baseline**	**6 months**	**24 months**	**Baseline**	**6 months**	**24 months**
Haemoglobin, mg/dL	10.0 ± 1.9	11.8 ± 2.3	12.5 ± 2.3	10.4 ± 1.8	12.5 ± 1.3	12.7 ± 2.0
BMI, kg/m^2^	18.2 ± 2.7	20.4 ± 2.7	21.8 ± 2.5	18.4 ± 2.7	20.0 ± 2.3	21.0 ± 2.4
Bilirubin, mg/dL	0.6 ± 0.2	0.7 ± 0.1	0.7 ± 0.1	0.6 ± 0.2	0.7 ± 0.1	0.7 ± 0.1
SGOT, IU/L	43.9 ± 26.4	34.1 ± 9.5	31.1 ± 11.1	42.6 ± 21.9	37.5 ± 21.5	34.4 ± 19.6
SGPT, IU/L	34.1 ± 23.6	32.6 ± 11.8	31.1 ± 11.6	36.0 ± 22.2	31.6 ± 17.9	36.8 ± 40.1

*BMI* Body mass index, *SGOT* Serum glutamic oxaloacetic transaminase, *SGPT* Serum glutamic pyruvic transaminase, *IU* International unit.

## Discussion

This open-label, randomised clinical trial demonstrated that, in HIV infected individuals with tuberculosis co-infection receiving rifampicin based ATT, there was no significant difference between twice daily nevirapine based ART and efavirenz based ART with respect to mortality, or immunological, virological and clinical response or side effect profile. There are multiple observational and retrospective studies comparing the clinical efficacy of nevirapine and efavirenz based ART in HIV/TB co-infected patients; however, results of this study were found contradictory to those [9,14-16]. The South African study [14] showed some difference between the two treatments, while the studies from Botswana [17] and Thailand [9,15] failed to demonstrate the difference. To the best of our knowledge, there are only two randomised control trials that have made this head to head comparison. However, one of these studies used 400 mg once a day nevirapine dose instead of the standard regimen of 200 mg twice a day [18]. The N2R trial by Manosuthi et al. showed no significant difference in the virological outcomes in patients receiving efavirenz or nevirapine based regimens [19].

Our results of comparable study of mortality, virological, clinical and immunological responses between the efavirenz and nevirapine groups are consistent with previous studies done in this patient sub-population [9,15-17,19]. In our study, there was a trend in increase in mortality in the nevirapine group as compared to that of the efavirenz group (19 · 4% vs. 14 · 7%); this difference, however, was not significant (P = 0 · 46). We also noted comparable rate of treatment failures in the two groups (30 · 9% in EFV group vs. 28 · 4% in NVP group, P = 0 · 75). The higher mortality observed in both the treatment groups as compared to other studies could be attributed to the long follow-up period of 2 years, and the fact that many of the patients enrolled in the trial had advanced tuberculosis (disseminated TB) and immunosuppression at the time of enrolment. This is supported by the fact that the majority of the deaths (87%) occurred in the early stage of the treatment in both groups.

Concerns have been raised about decreased plasma concentration and clinical efficacy of nevirapine in patients receiving rifampicin, due to induction of CYP450 enzyme [20]. In our study, the mean plasma concentration of nevirapine remained above 3 μg/ml except during the initial lead in period. Also, no correlation was found between the plasma drug levels and unfavourable outcomes. This is consistent with our previous study, where this lack of correlation between nevirapine blood levels and treatment outcomes in participants receiving rifampicin based ATT was demonstrated [10].

Our study demonstrated a favourable response in terms of cure and treatment completion rates (74 · 6% in NVP vs. 75% in EFV) of TB treatment in both the groups. During the 2 year follow-up, none of the patients who successfully completed their treatment relapsed. This rate of successful treatment is considered favourable and consistent with the data from older studies showing cure rates between 59 · 4% to 97 · 1% [21]. Though some studies have shown that thrice a week.

ATT might be sub-optimal in the first two months of the treatment in HIV-TB co-infections, we used the standard thrice a week DOTS regimens in accordance with the standard national guidelines available to us at the start of the trial [22]. It is possible that the differences in the ATT regimens accounted for the higher rate of failure in the nevirapine group in the study in South Africa by Boulle A. et al., which used once a day treatment [14]. It is still unclear at this time whether use of daily rifampicin will alter the efficacy of nevirapine in HIV-TB co-infected individuals. This remains an interesting question for future research, since very limited data are available to us at this point of time.

The overall rates of adverse drug events were low in both the treatment groups. Change in ART regimen was needed in few cases for adverse effects of one of the NRTI constituents, zidovudine or stavudine. No change in regimen was required with respect to the NNRTI component of ART. This is in contrast to the studies by Manosuthi et al. and Van Leth et al. which demonstrated a higher rate of hepatotoxicity in patients receiving nevirapine requiring change of regimen [9,11]. This study however included patients with hepatitis B and hepatitis C co-infections. It is possible that the exclusion of these patients in our study resulted in lower rates of hepatotoxicity. Also, some cohort studies have reported low and comparable levels of hepatotoxicity in efavirenz and nevirapine when these drugs were administered in combination with rifampicin; however, the levels were even higher in patients without TB [17,23]. The most commonly observed adverse event was mild skin reactions that did not require any treatment.

The important factor in our study was that this was a randomised control study while most of other studies comparing the efficacy of nevirapine and efavirenz in HIV/TB patients were observational studies. In addition, this study comprised a long follow-up period of 2 years which allowed us to study the outcome of TB in terms of failure and relapse. The longer follow-up period also enabled us to measure directly, the effect of the two regimens on mortality rather than just measuring virological suppression. Generalisability of the outcome measures is another strength, given the high disease severity in terms of degree of immunosuppression and severity of tuberculosis at baseline of our study population, which is typical for TB and HIV co-infection cohorts in resource constrained settings. Besides this, it also included correlation with nevirapine levels up to 6 months, and close monthly follow-up.

The study also had few limitations. Given the number of patients who completed the study follow-up at the end of the trial, the power of the study was less than 50%, and therefore was underpowered to detect a difference of less than 20% between the two groups. Many of the participants in the trial received stavudine, which has been recently phased out from the ART program in India. Another limitation was that the types of TB in the two groups differed significantly, and adjusting the types of TB did not alter the treatment outcomes significantly.

## Conclusions

In conclusion, the efficacy and safety of nevirapine based ART seemed to be comparable to that of efavirenz containing regimens. Owing to its lower cost and easy availability, nevirapine based ART could be an alternative in the resource limited settings in patients with HIV and tuberculosis co-infection.

## Competing interests

Authors declare that they have no competing interest.

## Authors’ contributors

SS provided inputs to the study design, helped in data analysis and interpretation, wrote the manuscript, and did final editing. RP, RCS, SD, SK, ME, SKS, SR, BBR and SV reviewed literature, and helped in interpreting data and writing the manuscript. TV conducted laboratory tests for nevirapine levels. HA and JCS conducted laboratory tests for CD4 cell count and plasma viral load. RMP performed data analyses. All authors read and approved the final manuscript.

## Pre-publication history

The pre-publication history for this paper can be accessed here:

http://www.biomedcentral.com/1471-2334/13/482/prepub
